# On an Instance of Extensive Malignant Disease of the Lungs and Heart, with Caries of the Ribs, Clavicle and Vertebræ

**Published:** 1848-09

**Authors:** D. W. Emmett

**Affiliations:** Surgeon, Darlington


*On an instance of extensive Malignant Disease of the Lungs and Heart, with caries of the ribs, clavicle and vertebræ.*—By D. W. Emmett, Esq. Surgeon, Darlington.—T. B., aged sixty-four, a weaver. came under medical treatment about the middle of April, 1846, complaining of pain, generally referrible to the cardiac region, accompanied by some degree of dyspnœa. His general appearance betokened ill health, his skin having a dirty, sallow appearance, and countenance being haggard and careworn. He had been gradually failing for some months—at times troubled with slight cough, unaccompanied by expectoration. On stripping him, and examining the chest, the upper part on each side was found flat. Over the cardiac region a little fulness was perceptible. On percussion, a marked difference in resonance was perceived, the whole of the left side emitting a peculiar dull sound. The right side was normal. On applying the stethoscope, a little crepitation existed under the right clavicle; otherwise the right lung appeared healthy. On applying it over the left lung, the respiratory murmur was completely inaudible, posteriorly, as well as in front and laterally. On the patient's coughing forcibly, and heaving a deep sigh, an indistinct noise was heard in one or two situations, corresponding with the division of the bronchi. On more minutely percussing the cardiac region, great dulness was observed. Sounds of the heart normal, but indicated great weakness. A simple course of treatment was adopted, with an opiate at bedtime. He experienced a slight amendment in his general health, but the "pain from the heart shoots down his left arm." Ordered, a stimulating liniment, with tincture of opium, to be rubbed into the chest night and morning, and the opiate to be repeated night and morning.

May 28th.—There was marked disturbance in the heart's action; breathing short, accompanied by intense pain; could not bear examination. Calomel and opium gave considerable relief; and as this appeared due to the opium, he was put entirely under the influence of that drug, and of morphia and hyoscyamus. Sounds of the heart remained normal; but attacks of syncope occurred, and this symptom soon became alarming, from its frequent occurrence and long duration. It would be tedious to report his progress minutely; he continued in a state of great suffering, in spite of opiates. The pain in the cardiac, subclavian, and scapular regions, became more intense; and when percussed, he experienced, apparently, electric shocks, his whole frame starting with agony. The cellular tissue over these regions became œdematous, though giving more the idea of emphysema than œdema. On the right side moist and indistinct râles were heard, the respiration was slow, but a small portion of lung seemed to be acting. Towards the last, the manual examinations were omitted, as the slightest tap distressed him. The faintings became more frequent, the pain more agonizing, the difficulty in swallowing greater, until death relieved him from his sufferings, on the 30th of July.

*Post-mortem examination, twelve hours afterwards.*—The examination was made by C. Boromar, Esq., of Leicester, and myself. The body was considerably emaciated, and decomposition already commenced. On raising the sternum, the left side of the chest was found densely filled with a hard, unyielding substance; the right lung very much collapsed. The heart was firmly pressed against the theoracic parietes, and the pericardium was adherent. On endeavouring to remove the contents of the thorax, we found the whole of the left lung was converted into a mass of cretaceous tubercular matter, firmly adherent, in the whole extent, to the costa. The right lung presented a remarkable difference, breaking up like so much moist tissue paper, and covering the hands with a black fluid, having every appearance of being a melanotic production; but a very small portion of the inferior part of this lung was capable of carrying on respiration. On opening the perdicardium, the heart was found about the natural size, though rather atrophied. The valves were quite healthy; lining membrane of heart dark, as if saturated with black, putrid blood; substance of heart very soft. In the wall of the left ventricle, Mr. Boromar discovered a large scirrhous tubercle, or rather tumour, about the size of a walnut, of a cartilaginous hardness, the centre not so firm as the exterior. On further removing lumps of the strangely metamorphosed left lung, we found the first, second. third, and fourth ribs quite carious and brittle; the under surface of the left clavicle was sharp, rough, and in the same condition. We found the vertebræ diseased to a much greater extent than I should have thought likely to exist, without having caused more characteristic symptoms to exist. The first and second dorsal vertebræ were so perfectly carious, that the finger could be readily placed upon the theca of the spinal cord. None of the other viscera showed any marked disease. The brain was not examined. The œsophagus, as expected, was found flattened, and partially obstructed, by the pressure of the diseased mass, fully accounting for the difficulty experienced in swallowing.

I was much puzzled, at first, with this case. Everything showed that great structural change had occurred, but of what nature could not be so easily detected. The stethoscope was of use, though negatively, assisting considerable in deciding *what* the disease was *not*. Hydrothorax, hepatization, aneurism, &c., where all dismissed, and the case considered to be one of some rare organic alteration; and judging from concomitant symptoms, very probably malignant. The prognosis and treatment, after this decision, became readily determined. He was consequently placed as much as justifiable under the influence of opiates, morphia, and compound spirit of sulphuric ether. It is gratifying to reflect that the old veteran (he had fought at Waterloo) was not tortured by a meddlesome and experimental mode of treatment, as the result proved that all such must have been attended with disappointment. We were obliged to discontinue the examination, Mr. Boromar having an engagement elsewhere, and my attention being directed to a wound in the finger, caused by a spicula of carious bone.—*London Lancet.*

